# Numerical investigation of knee prosthesis stresses in daily activities: Insight into knee rehabilitation and Creation of a new optimal model

**DOI:** 10.1016/j.heliyon.2024.e37657

**Published:** 2024-09-12

**Authors:** Seyedhamidreza Emadiyanrazavi, Shahrokh Shojaei

**Affiliations:** Department of Biomedical Engineering, Central Tehran Branch, Islamic Azad University, Tehran, Iran

**Keywords:** Total knee arthroplasty, von mises stress, Finite element method (FEM), Knee prosthesis

## Abstract

Total knee arthroplasty (TKA) is a cornerstone in addressing knee joint disorders, significantly enhancing patients' quality of life. However, despite technological advancements, a comprehensive understanding of the dynamic stresses experienced by knee prostheses during daily activities, particularly under rehabilitation interventions, remains elusive. This study aims to bridge this gap by employing numerical simulations and finite element analysis to elucidate these dynamic stresses and their interaction with rehabilitation protocols.

A real-life knee replacement prosthesis model was meticulously constructed through coordinate measuring and 3D scanning, facilitating detailed finite element analysis in ANSYS Workbench version 17.1. Two distinct boundary conditions and loading scenarios were applied, with comparisons made between linear and nonlinear material assumptions. The simulation results using these different boundary condition methods revealed minimal differences. Specifically, at a knee angle of 0°, the relative stress error rate between the two boundary condition types was approximately 1 % (1.11 MPa and 1.099 MPa, respectively). At 15° and 90°, the error rates were 1.9 % and 0.56 %, respectively (10.275 MPa and 10.078 MPa at 15°; 10.275 MPa and 10.078 MPa at 90°). Given these minimal differences, the first type of boundary condition was adopted for the subsequent scenarios to enhance convergence efficiency in the analysis.

Moreover, comparative analyses between linear and nonlinear material behaviors demonstrated acceptable agreement, offering insights into potential efficiency gains in simulation methodologies. Building on this foundation, an optimized tibial model was proposed, incorporating geometric alterations to the tray. Quantitative assessments revealed significant reductions, with von Mises stress decreasing by 23.35 % and equivalent strain by 17 % at a knee angle of 140°. Further evaluations at varying angles, including 60°, consistently showed positive influences on stress and strain. These quantitative findings not only contribute valuable insights into the mechanical behavior of knee prostheses but also provide tangible evidence for the efficacy of linear material behavior assumptions. The proposed optimized model exhibits promising potential for enhancing the design and performance of knee prostheses, particularly under critical loading conditions.

In conclusion, these results underscore the importance of a nuanced understanding of knee prosthesis behavior during rehabilitation, offering a quantitative foundation for refining existing designs and informing the development of next-generation prostheses.

## 1 Introduction

Knee prostheses have significantly transformed orthopedic interventions, becoming essential for individuals with knee joint disorders such as osteoarthritis or post-traumatic injuries [[1], [2], [3]]. The integration of advanced materials and engineering techniques has enhanced the durability and stability of these prostheses, leading to improved mobility and quality of life for many patients [4,5]. Despite these advancements, there remains a significant gap in understanding the dynamic stresses that knee prostheses experience during daily activities, particularly when subjected to rehabilitation interventions [[6], [7], [8]]. This lack of knowledge can affect the long-term success and performance of knee prostheses, emphasizing the need for further research in this area.

A comprehensive review of the existing literature reveals the remarkable progress achieved in knee prosthesis technology. These advances aim to replicate the natural biomechanics of the knee joint, enabling patients to enjoy a broader range of motion, improved functionality, and an enhanced quality of life [[9], [10], [11], [12]]. Over the years, various studies have delved into the intricate aspects of knee prostheses, encompassing the materials used, surgical techniques, and design considerations [[13], [14], [15], [16], [17]]. These investigations have substantially enriched our understanding of knee replacement surgery, leading to more durable and reliable prosthetic devices [[18], [19], [20]].

Several notable studies have explored the mechanical properties and structural design of knee prostheses. For instance, a study by Suwattanarwong Phanphet et al. (2017) enhanced the design standards of above-knee prostheses by Thailand's Prostheses Foundation, addressing fatigue failures in specific components during normal cyclic loading. Finite element simulations and Morrow's approach were used to model fatigue life predictions, resulting in an optimized design that successfully met stress, deflection, and fatigue life criteria [18]. Similarly, Saran Keeratihattayakorn et al. (2019) introduced a cost-effective design using commonly available components to address the challenges posed by expensive commercial hydraulic knee prostheses. Their prototype demonstrated efficacy in preventing knee flexion during the stance phase and withstood a flexion torque of 60 N-m, making it suitable for low-income regions [21].

FM Kadhim et al. (2020) evaluated four prosthetic knee designs for above-knee amputees, identifying the polycentric knee with geometric locking and hydraulics as the top performer. This design showed minimal differences in ground reaction force between healthy and prosthetic limbs, favorable interface pressures, and optimal gait symmetry [22]. Additionally, Affatato et al. (2019) investigated the tribological performance of antibiotic-impregnated knee spacers under dynamic loading conditions, suggesting their potential efficacy as wear-resistant temporary implants [23]. Lalitha Amirapu et al. (2022) employed finite element analysis to assess the structural integrity of a polymeric-based knee implant reinforced with a nanodiamond nanocomposite spacer, endorsing the suitability of the composite material for knee arthroplasty applications [24].

In another study, a biomechanical analysis of different levels of constraint in total knee arthroplasty (TKA) during daily activities was conducted. The study compared various prosthesis designs, revealing significant differences in contact areas and stress distribution at the interface between prosthetic components, although tibial bone stress remained relatively homogeneous [25]. A static simulation of polycentric prosthetic knees by analyzing stress distribution and overall deformation under vertical loading demonstrated that the prosthetic knee could withstand enough stress and strain to comply with ISO 10328:2006 structural requirements [26]. Research by Mohd Afzan Mohd Anuar and Mitsugu Todo investigated the mechanics of posterior-stabilized TKA during daily activities, highlighting the relationship between prosthesis design and stress conditions. Their study emphasized the importance of conforming interfaces for good wear resistance and reduced surface distortion [27]. Additionally, in vivo measurements of contact stresses during daily activities after knee arthroplasty demonstrated that high-flexion activities generated significantly higher stresses compared to walking and stair climbing. This underscores the need for "high-flexion" designs that preserve contact area at high flexion angles [28]. Finally, a study on the design and analysis of polycentric prosthetic knees aimed to improve existing designs by conducting an engineering failure analysis. The modified design outperformed the existing knee prosthesis in terms of stress distribution, deformation, and fatigue strength, indicating a safe and stable design with a predicted lifespan of at least ten years [29].

These studies collectively highlight the advancements and ongoing challenges in knee prosthesis design and functionality. They also underscore the necessity of further research to understand the dynamic stresses experienced by knee prostheses during rehabilitation exercises. This understanding is crucial for optimizing rehabilitation protocols and developing next-generation knee prostheses that better accommodate the demands of rehabilitation and improve patient satisfaction.

Despite significant advancements in knee prosthesis technology and design, a critical gap remains in our understanding of the dynamic stresses experienced by knee prostheses during rehabilitation exercises. Most existing studies have focused on the static mechanical properties and structural integrity of knee implants under typical daily activities. However, the specific impact of rehabilitation interventions on the mechanical behavior of knee prostheses has not been thoroughly investigated. This lack of knowledge hinders the optimization of rehabilitation protocols and the development of more robust and adaptive prosthetic designs. This study aims to investigate the dynamic stresses experienced by knee prostheses during various rehabilitation exercises using numerical simulations and finite element analysis. The research seeks to analyze how different rehabilitation activities affect the stress distribution within knee prostheses and their interfaces. The ultimate goal is to develop and propose an optimized knee prosthesis model that can better withstand the demands of rehabilitation exercises. The underlying hypotheses are that rehabilitation exercises impose significant dynamic stresses on knee prostheses, differing from those experienced during regular daily activities, and that understanding these stresses will reveal critical insights into the mechanical behavior of knee prostheses. This knowledge is expected to inform the design of more durable and adaptive implants, leading to improved patient outcomes by enhancing the reliability and functionality of the implant during rehabilitation.

Understanding the dynamic stresses exerted on knee prostheses during rehabilitation is crucial for several reasons. Firstly, it can lead to the improvement of prosthesis design, ensuring that implants are better equipped to handle the specific demands of rehabilitation activities. This can result in enhanced durability and longevity of the prosthetic devices, reducing the need for revisions and improving patient satisfaction. Secondly, insights gained from this study can inform the development of more effective rehabilitation protocols. By tailoring rehabilitation exercises to account for the mechanical behavior of the prosthesis, clinicians can optimize recovery processes, minimize complications, and improve overall functional outcomes for patients. Lastly, this research holds the potential to influence the design of next-generation knee prostheses. By incorporating findings related to dynamic stress responses, manufacturers can create implants that not only meet the static demands of daily activities but also the dynamic challenges posed by rehabilitation. This could lead to a new standard in prosthetic design, ultimately improving the quality of life for patients undergoing knee arthroplasty. Therefore, this study aims to fill a critical gap in the current body of knowledge, with the potential to significantly impact both clinical practices and prosthetic design, thereby enhancing patient outcomes and satisfaction.

## 2 Materials and methods

### 2.1 General Steps of the study

To initiate the study, a real-life knee replacement prosthesis suitable for a patient weighing approximately 80 kg was selected. Using a coordinate measuring machine—a contact-based 3D scanner—the coordinates of the prosthesis geometry were obtained in a point cloud format. Subsequently, utilizing SolidWorks software (version 2017), a volumetric and analyzable model was constructed [[30], [31], [32]].

The created model was then subjected to numerical simulation using the finite element method in ANSYS Workbench software version 17.1. In the simulation phase, two types of boundary conditions were applied to analyze the stress-strain behavior of the knee prosthesis during critical positions in daily activities. The results obtained from linear and nonlinear simulations were compared to assess the behavior of the knee prosthesis materials. Based on these analyses, a modified tibial model was examined, and an optimized tibial model was proposed to reduce the incoming stresses.

### 2.2 Geometric modeling of knee prostheses

**Fig. 1:**
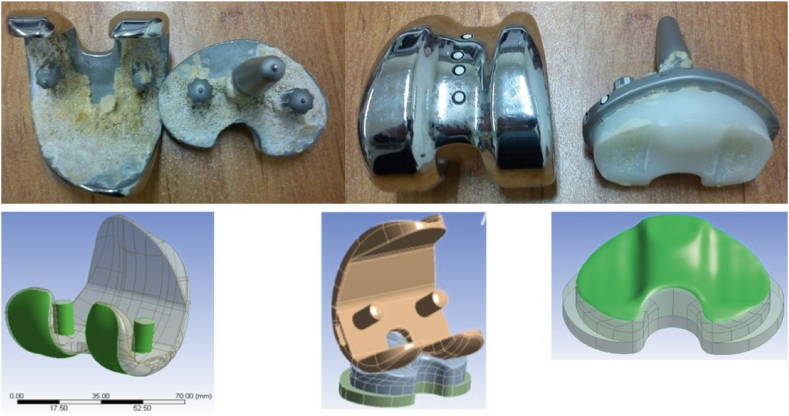
Geometric specifications comparison between real-life prosthetic and simulated model, illustrating congruence.

To accurately represent the knee prosthesis, the coordinates defining the geometry of the prosthesis were obtained in a point cloud format using a coordinate measuring machine. The resulting point cloud model needed to be converted into a useable 3D model for simulation purposes. Hence, employing SolidWorks software (version 2017), a volumetric and analytically accessible model was carefully created. During the editing stage of the prosthetic components, emphasis was placed on edge refinement and removal of sharp features to increase model accuracy and simulation efficiency. Fig. 1 illustrates the geometric specifications of both the real-life prosthetic geometry and the generated model, showcasing the comparison between the actual and the simulated geometry. The specific prosthesis used in our study is the Zimmer Biomet knee prosthesis. Below are the details added to the manuscript.

⁃ Manufacturer: Zimmer Biomet (United States)
⁃ Model Number: NexGen® Complete Knee Solution
⁃ Structural Conformity: The prosthesis conforms to relevant international standards for knee joint prostheses.

### 2.3 Finite element model and impact conditions

**Fig. 2:**
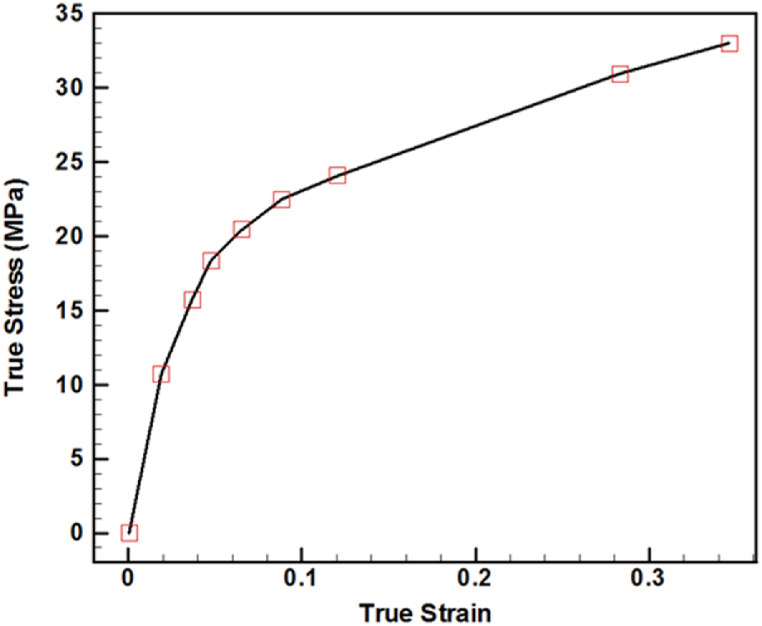
Stress-strain curve for UHMWPE, the material used in elastic modeling of the tibial tray [35].

The mechanical properties employed in the knee prosthesis FEM are presented in Table 1 [33]. The femoral and tibial tray were modeled using Cobalt-Chrome alloy in an elastic form. Additionally, the tibial tray was modeled with Ultra-High Molecular Weight Polyethylene (UHMWPE) in an elastic form. The stress-strain curve for UHMWPE can be observed in Fig. 2 [34].

**Table 1:** Mechanical properties used in knee prosthesis FEM [35].

Components	Behavior	Material Name	Density (kg/m^3^)	Young's Modulus	Poisson's Ratio
Femoral	Elastic	Cobalt-Chrome Alloy	4620	200 GPa	0.3
Tibial Tray	Elastic	Cobalt-Chrome Alloy	4620	200 GPa	0.44
Tibial	Elastic	UHMWPE	920	1.1 GPa	0.44

**Fig. 3:**
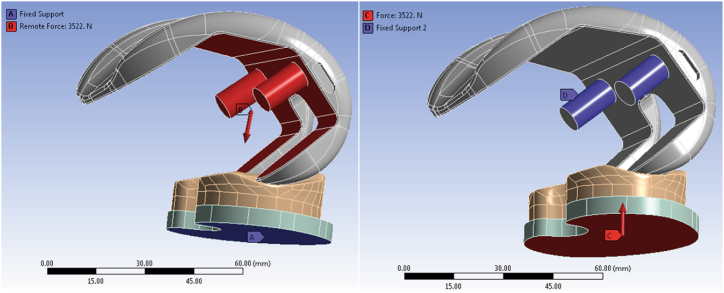
Two boundary conditions illustrated. Method 1: Fixed femoral portion, force on tibial tray. Method 2: Fixed tibial tray, force at joint center.

Two distinct boundary conditions were employed. In the first method, a portion of the femoral component remained fixed while a perpendicular force was applied to the tibial tray's surface. In the second method, the tibial tray's surface was fixed, and a force was applied at the joint center perpendicular to the femoral surfaces along the tibial surface. The boundary conditions for both methods are shown in Fig. 3. The results from these two methods were presented and compared. Table 2 provides the values of applied forces at different positions [36].

**Table 2:** Applied forces on the tibial surface in various positions [[36], [37], [38]].

Activity	Knee Angle (°)	Applied Force ( × Patient's Weight)	Perpendicular Force on Tibial Surface (N)
Standing	0	0.5	450
Walking	15	3–3.5	2747
Going up and down stairs	45–60	3.8–4.3	3375
Rising from a chair	90	3.3	2590
Standing on both knees	135	4.5	3532
Squatting	140	5–5.6	4395

**Fig. 4:**
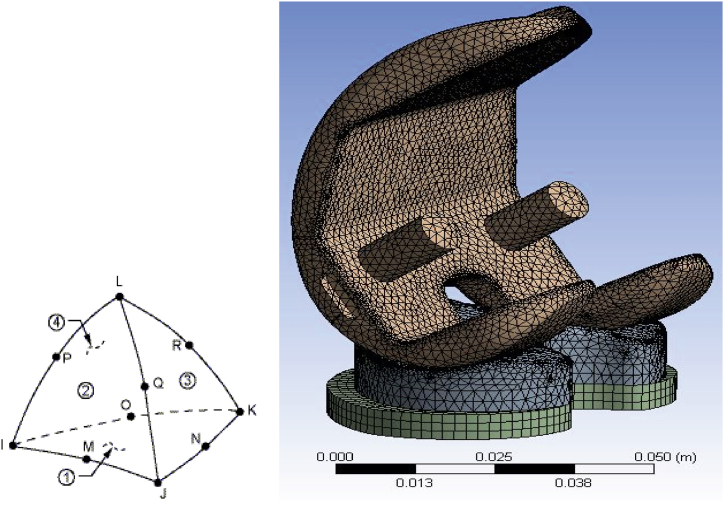
Left: A depiction of the element type used in the finite element model (Solid 187). And Right: A visual representation of the meshing of the knee prosthesis model.

**Fig. 5:**
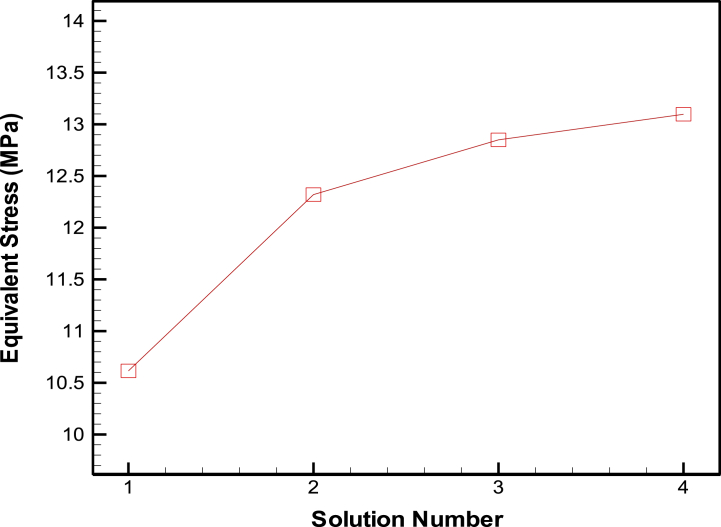
Insensitivity of von Mises stress to element size of knee prosthesis.

For knee prosthesis FEM meshing, a Solid 187 element type was utilized, which includes 32,837 elements and 66,030 nodes, as shown in Fig. 4. The sensitivity to the number of elements was checked and the results with this number of elements are independent of meshing (Fig. 5). Furthermore, it's noteworthy that with this specific mesh configuration, we achieved convergence of von Mises stress at a knee angle of 60°, with a resulting stress of 12.49 MPa and a deviation of 4.6 %. This convergence was attained with a mesh containing 120,357 elements.

## 3 Results

The results obtained from finite element simulation of the knee prosthesis using ANSYS Workbench software version 17.1 with the application of two different boundary conditions were extracted. Considering the material properties used in various parts of the prosthesis and the fact that the most vulnerable component in the knee prosthesis under study is the tibial tray, only the stress and strain analysis of the tibial component at various knee angles were presented as the primary focus of this investigation [[33], [34], [35], [36], [37], [38], [39], [40]].

The stress analysis in this study was conducted using the Max. von Mises Stress criteria. This criterion was selected due to its established utility in assessing the yielding or failure of materials under complex loading conditions, which are typical in biomechanical applications such as knee prostheses subjected to varying daily activities and rehabilitation scenarios. The Max. von Mises Stress criterion accounts for both tensile and compressive stresses and is effective in predicting material failure under multiaxial loading, which is common in joint biomechanics. By considering the combined effect of different stress components, this criterion allows for a comprehensive analysis of potential failure risks in knee prostheses. Furthermore, the Max. von Mises Stress criterion aids in identifying critical stress regions that may lead to material failure or fatigue, thus informing design improvements and rehabilitation strategies.

### 3.1 Comparison of simulation results with two boundary condition methods

**Fig. 6:**
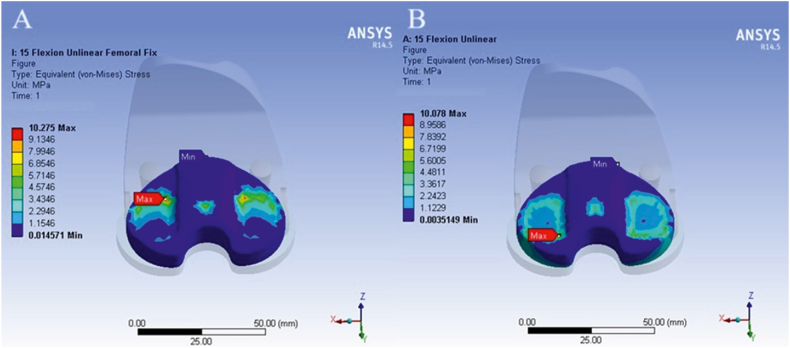
Contour of von Mises stress in the tibial component with the application of the first (A) and second (B) boundary condition methods at a knee angle of 15°.

**Fig. 7:**
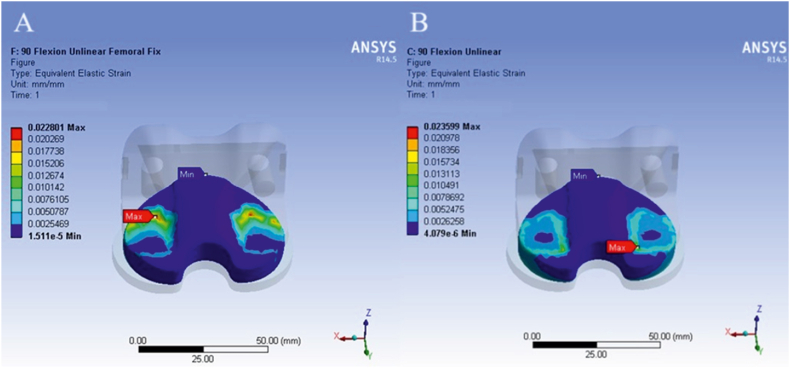
Contour of equivalent strain in the tibial component with the application of the first (A) and second (B) boundary condition methods at a knee angle of 90°.

Fig. 6, Fig. 7 compare the results obtained from simulations using two different boundary condition methods. Contours of von Mises stress and strain in the tibial component of the knee prosthesis are presented as examples of the numerical simulation results.

According to Table 3, which presents the simulation results using two different boundary condition methods, the differences observed were minimal. Specifically, at a knee angle of 0°, the relative stress error rate between the first and second boundary condition types was approximately 1 %. At a knee angle of 15°, the error rate was 1.9 %, and at 90°, it was 0.56 %. Given these minimal differences, the first type of boundary condition was applied in the remaining scenarios to enhance convergence efficiency in the analysis.

**Table 3:** Comparison of results obtained from simulations using two boundary condition methods.

Knee Angle (°)	Boundary Type	Equivalent Strain	Relative Strain error rate (%)	Stress (MPa)	Relative Stress error rate (%)
0	First	0024/0	3/4	11/1	1
0	Second	0023/0		099/1	
15	First	0210/0	4/3	275/10	9/1
15	Second	0203/0		078/10	
90	First	0228/0	7/2	282/11	56/0
90	Second	0235/0		346/11	

### 3.2 Comparison of simulation results with linear and nonlinear material behavior assumptions

In this section, we present the results obtained from simulations assuming both linear and nonlinear material behaviors. The objective was to reduce convergence time in nonlinear simulations by considering the elastic behavior of the tibial tray based on the results presented. Previously, all results were derived assuming nonlinear material behavior. Now, we compare the results of simulations assuming linear material behavior for knee angles of 15° and 90° using the first type of boundary condition with the previous nonlinear results, as shown in Table 4.

**Table 4:** Comparison of results obtained from simulations assuming linear and nonlinear material behaviors.

Knee Angle (°)	Material Behavior	Equivalent Strain	Relative Strain error rate (%)	Stress (MPa)	Relative Stress error rate (%)
15	Nonlinear	0210/0	44/1	275/10	46/2
15	Linear	0207/0		028/10	
90	Nonlinear	0228/0	2/6	282/11	16/0
90	Linear	0213/0		3/11	

At a knee angle of 0°, the relative strain error rate between nonlinear and linear material behaviors is 1.44 %, and at 90°, it is 6.2 %. Additionally, the relative stress error rates at knee angles of 0° and 90° are 2.46 % and 0.16 %, respectively.

These results indicate that the simulations assuming linear and nonlinear material behaviors of the tibial tray exhibit acceptable agreement. Therefore, to improve convergence efficiency while retaining the elastic state of the tibial tray, assuming linear material behavior can be employed.

### 3.3 Simulation results assuming linear material behavior in different positions

**Fig. 8:**
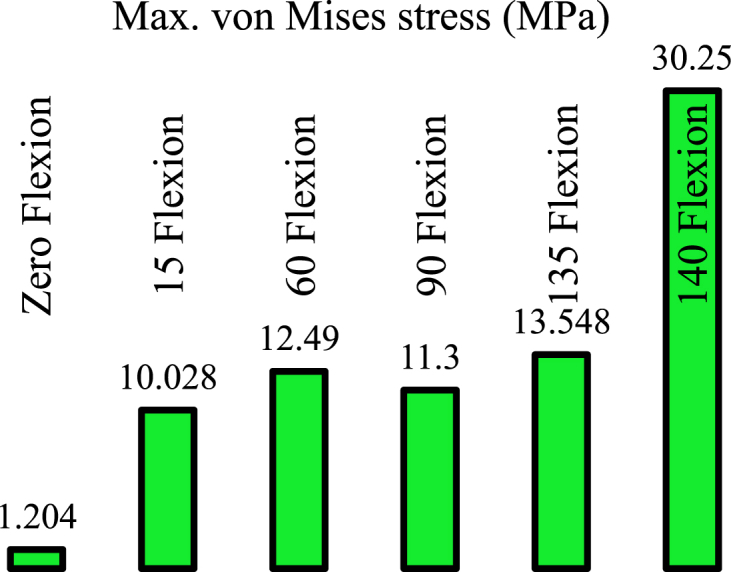
Equivalent von Mises stress in the tibial component assuming linear material behavior in different loading positions.

**Fig. 9:**
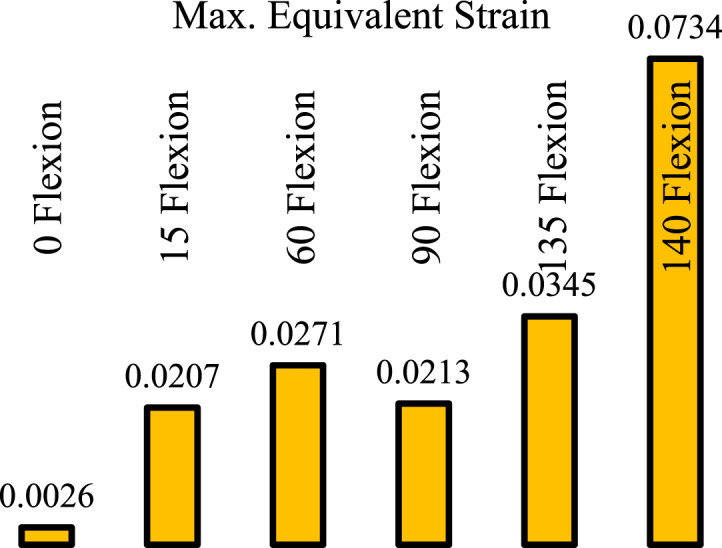
Equivalent strain in the tibial component assuming linear material behavior in different loading positions.

This section presents the critical results of the knee prosthesis simulation, focusing on von Mises stress and equivalent strain in the tibial component under linear material behavior at the most critical loading positions, specifically 135 degrees of flexion. Fig. 8, Fig. 9 illustrate the equivalent von Mises stress and strain in the tibial component for various loading positions under the assumption of linear material behavior. The equivalent von Mises stress in the tibial component was recorded as follows: 1.204 MPa at zero flexion, 10.028 MPa at 15° flexion, 12.49 MPa at 60° flexion, 11.3 MPa at 90° flexion, 13.548 MPa at 135° flexion, and 30.25 MPa at 140° flexion. Similarly, the equivalent strain values were 0.0026, 0.0207, 0.0271, 0.0217, 0.0345, and 0.0734 at 0, 15, 60, 90, 135, and 140° flexion, respectively. These results demonstrate the distribution of von Mises stress and equivalent strain in the tibial component across various flexion angles, highlighting the most critical loading positions. The assumption of linear material behavior provides valuable insights into the mechanical performance of the knee prosthesis under different loading conditions, which is essential for optimizing prosthesis design and improving patient outcomes.

### 3.4 Validation results

**Fig. 10:**
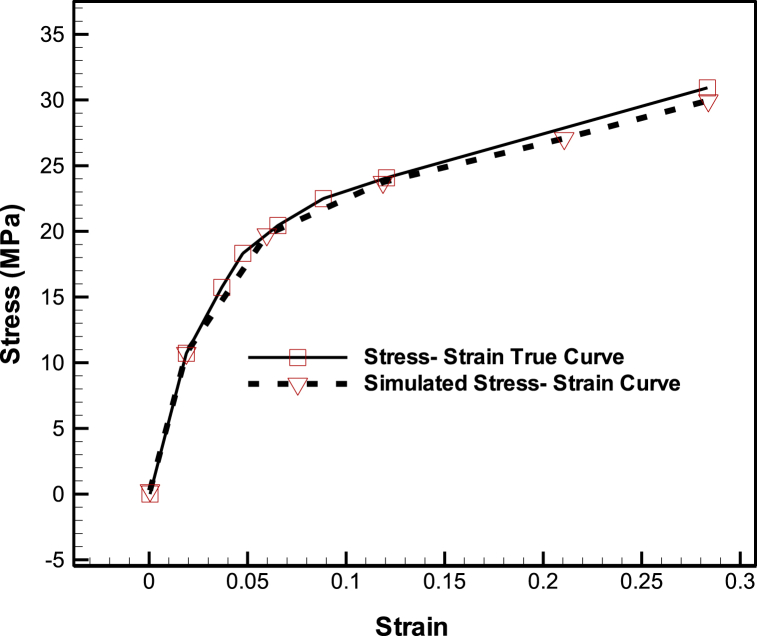
Comparison of stress-strain true curve and stress-strain curve extracted from knee prosthesis simulation.

In Fig. 10, the stress-strain true curve and the stress-strain curve extracted from the simulation of the knee prosthesis are compared. The result of the comparison indicates that the simulation results are in good agreement with the experimental data provided by J. Shi [35]. Also, the sensitivity to the number of elements was checked and the results are independent of meshing.

### 3.5 Optimized model of tibial and tibial tray

Considering the contours presented in the previous section, plastic deformation occurs at a knee angle of 140° with an equivalent strain of approximately 0.0734 [35]. In this section, an attempt has been made to reduce the applied stresses by modifying the geometric model of the tibial and tibial tray in the knee prosthesis under study.

In previous studies, including Mallesh et al., 2012, investigated the effects of tibial tray radius and knee flexion angles on joint stresses during finite element modeling and analysis of knee prostheses. According to their research, increasing the radius of the tibial tray leads to an increase in von Mises stress [41]. The reason for this increase is the larger contact surface, which leads to higher local pressure and stress. Simultaneously, increased knee flexion angles also contribute to increased von Mises stress and shear stress due to the expanded contact surface. It is noteworthy that the profile of the contact surface of the knee prosthesis significantly influences the mechanical behavior of the knee joint. However, adhering to the natural shapes of the femur and tibia bones imposes constraints on altering the geometric profile of the contact surface. Consequently, it was deemed necessary to optimize the mechanical characteristics of the prosthesis material by modifying the tray's geometry.

**Fig. 11:**
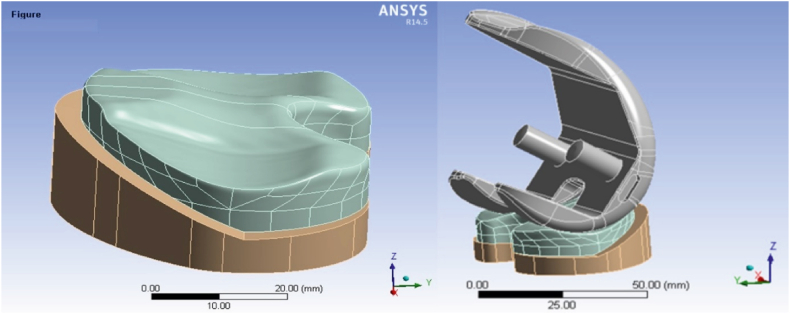
Presentation of a novel tibial and tibial tray model, highlighting modifications to the prosthesis tray's smooth surface with two inclined surfaces at varying angles.

In this regard, a new model of the tibial and tibial tray was created, as shown in Fig. 11. In this model, the smooth surface of the prosthesis tray was altered to two inclined surfaces with different angles. Table 5 demonstrates the effect of changing the angles of the lateral and medial surfaces of the prosthesis tray on the stress and strain in the tibial component. It is clear that changing the angles of the surfaces created in the model leads to a change in the equivalent stress and strain.

**Table 5:** Comparison of results obtained from simulations with linear and nonlinear material behaviors.

Lateral Surface Angle (°)	Medial Surface Angle (°)	Maximum Stress (MPa)	Maximum Elastic Strain
**3**	20	18/23	0609/0
**3**	15	96/27	064/0
**3**	25	41/27	062/0
**1**	20	82/28	063/0
**1**	15	53/29	056/0
**1**	25	82/27	056/0
**5**	20	07/28	062/0
**5**	15	01/33	066/0
**5**	25	82/29	059/0
**0**	15	50/26	059/0
**0**	18	12/32	058/0
**0**	12	01/29	064/0
**2**	15	46/26	061/0
**2**	18	31/26	057/0
**2**	12	74/28	060/0

**Fig. 12:**
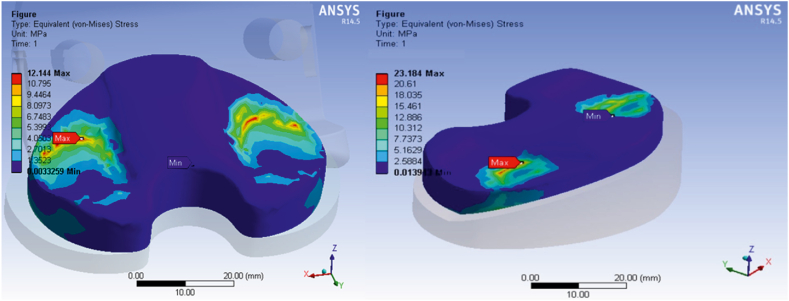
Stress contours of the presented model simulated at knee angles of 60 and 140°. Comparison with the original knee prosthesis model results.

The presented model was simulated at two knee angles, 60 and 140°, and the stress contours are illustrated in Fig. 12. The results of the new model were compared with the results of the original knee prosthesis model.

**Fig. 13:**
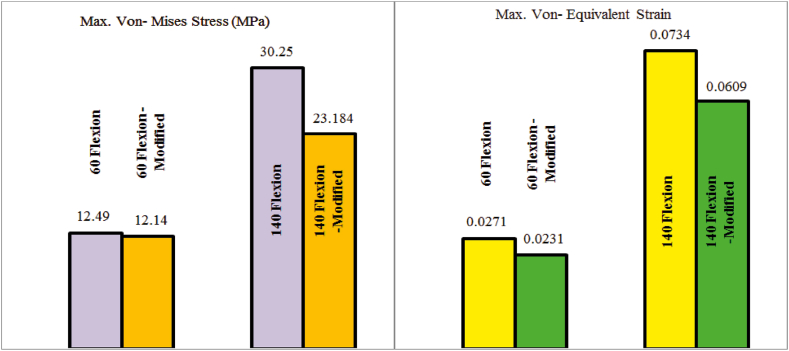
Maximum von Mises stress (Left) and Maximum equivalent strain (Right).

Based on Fig. 13, the equivalent von Mises stress in the tibial component decreased by 23.35 % and the equivalent strain decreased by 17 % in the new model at a knee angle of 140° compared to the original prosthesis model. Additionally, to ensure this positive effect in other scenarios, simulation results were presented for a knee angle of 60°, showing a reduction of 8.2 % in stress and 14.76 % in strain. Considering the distance of other angles from the plastic state, the modification of the model is generally perceived as a positive influence under critical loading conditions.

## 4 Discussion

The results of this study provide quantitative insights into the dynamic stresses encountered by knee prostheses during daily activities and rehabilitation, offering valuable data for advancing computational biomechanics research. By employing numerical simulations and finite element analysis, we investigated the biomechanical behavior of knee prostheses, focusing on the dynamic stresses experienced during typical daily activities and rehabilitation exercises. Our findings highlight the critical importance of incorporating rehabilitation interventions in biomechanical studies, as these interventions can significantly influence the long-term performance and outcomes for individuals with knee joint disorders. This study underscores the necessity of detailed biomechanical analysis to enhance the design and efficacy of knee prostheses, ultimately improving patient outcomes.

The comparison of simulation results using two different boundary condition methods demonstrated minimal differences, indicating high convergence efficiency in the analysis. Specifically, at a knee angle of 0°, the relative stress error rate between the first and second boundary condition types was approximately 1 %. At a knee angle of 15°, the error rate increased slightly to 1.9 %, and at 90°, it decreased to 0.56 %. These minimal differences underscore the reliability and robustness of the first boundary condition method. Consequently, the first type of boundary condition was applied in all subsequent scenarios to ensure optimal convergence efficiency throughout the analysis [39,41].

The comparison between linear and nonlinear material behavior assumptions for the tibial tray demonstrated acceptable agreement, suggesting that assuming linear material behavior can be employed to enhance convergence efficiency without compromising accuracy. The objective was to reduce convergence time in nonlinear simulations by considering the elastic behavior of the tibial tray, based on the presented results. Previously, all simulations assumed nonlinear material behavior. We now compare the results of simulations assuming linear material behavior at knee angles of 15° and 90°, using the first type of boundary condition, with the previous nonlinear results, as shown in Table 4. At a knee angle of 0°, the relative strain error rate between nonlinear and linear material behaviors is 1.44 %, and at 90°, it is 6.2 %. Additionally, the relative stress error rates at knee angles of 0° and 90° are 2.46 % and 0.16 %, respectively. These results indicate that simulations assuming linear and nonlinear material behaviors of the tibial tray exhibit acceptable agreement. Therefore, to improve convergence efficiency while maintaining the accuracy of the elastic state of the tibial tray, assuming linear material behavior can be effectively employed [[39], [40], [41]].

The stress and strain analysis of the tibial component under different knee angles provided crucial insights into the mechanical behavior of knee prostheses. The contours of von Mises stress and equivalent strain, particularly at critical knee angles, revealed patterns that can inform prosthesis design modifications [35].

The comparison of simulation results with linear and nonlinear material behaviors demonstrated that assuming linear material behavior for the tibial tray can be employed without compromising accuracy. This finding contributes to the efficiency of numerical simulations, reducing convergence time while retaining the elastic state of the tibial tray [35,40].

One of the significant contributions of this study is the proposal of an optimized tibial and tibial tray model based on geometric modifications. The inclination of the prosthesis tray's surfaces at varying angles demonstrated a positive influence, reducing equivalent stress and strain, particularly at critical knee angles (e.g., 140°). This modification, inspired by previous studies on tibial tray radius and knee flexion angles, showcases the potential for refining prosthesis designs to mitigate stress concentrations and enhance overall performance.

In previous research, such as the study by Mallesh et al., in 2012, the impact of tibial tray radius and knee flexion angles on joint stresses during finite element modeling and analysis of knee prostheses has been extensively explored [41]. Their findings indicated a direct correlation between the increase in the tibial tray radius and the rise in von Mises stress levels. This phenomenon can be attributed to the larger contact surface area, which results in elevated local pressure and stress within the joint. Similarly, variations in knee flexion angles were found to amplify von Mises stress and shear stress due to the expanded contact surface.

**Fig. 14:**
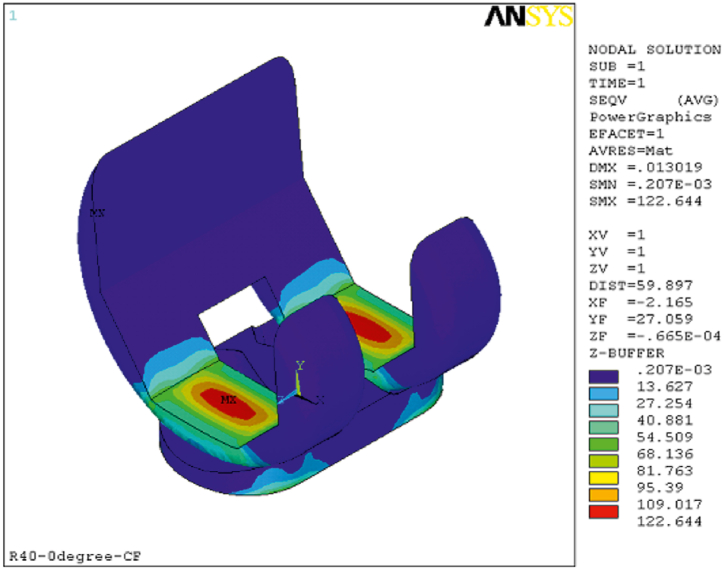
Von Mises stress distribution for a sagittal radius of 40 mm and knee angle of 0°.

**Fig. 15:**
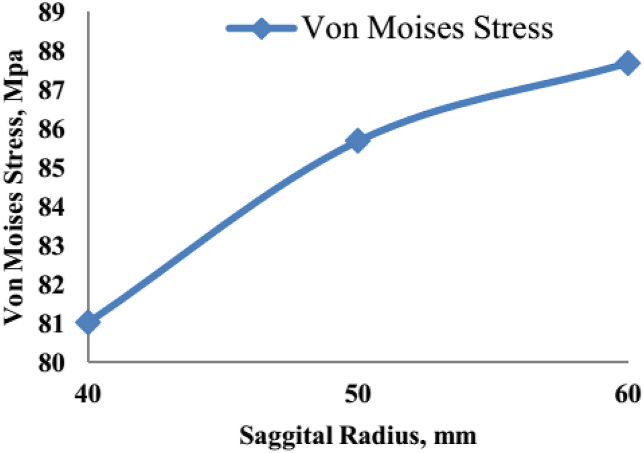
Variation in von Mises stress for different values of the sagittal radius.

Our study corroborates these findings, as depicted in Fig. 14, which illustrates the distribution of von Mises stress for a sagittal radius of 40 mm and a knee angle of 0°. Additionally, Fig. 15 showcases the alterations in von Mises stress for different sagittal radius values. Consistent with Mallesh et al.'s research, our results indicate a notable increase in von Mises stress with an escalation in the sagittal radius. Notably, this increase is independent of the material's gender.

The rise in von Mises stress with an increasing sagittal radius can be attributed to the augmented contact surface area, resulting in heightened support pressure and stress levels. Conversely, the decrease in shear stress with an increasing sagittal radius can be attributed to the alleviation of stress concentration at the joint surface.

Furthermore, our findings indicate that increasing the knee angle leads to a concurrent rise in both von Mises stress and shear stress due to the expanded contact surface area. This observation underscores the complex interplay between geometric factors and mechanical stresses within knee prostheses, highlighting the need for meticulous consideration of design parameters in optimizing prosthesis performance and longevity.

The insights gained from this study have implications for the future design of knee prostheses. The optimized model, with its reduced stress and strain under critical loading conditions, suggests a direction for improving the mechanical characteristics of knee prostheses. This may contribute to the development of next-generation prostheses that better adapt to the diverse demands of daily life, especially during rehabilitation.

⁃ While this research provides valuable insights into the biomechanics of knee prostheses during daily activities and rehabilitation, it is important to acknowledge certain limitations that may impact the interpretation and generalization of the findings.
⁃ Simplified Modeling Assumptions: The simplification of material behaviors, especially the assumption of linear material behavior for the tibial tray, may not fully capture the complexities of real-world prosthetic materials.
⁃ Boundary Conditions and Loading Scenarios: The study focused on two specific types of boundary conditions and loading scenarios to analyze the stress-strain behavior of knee prostheses. While these scenarios aimed to simulate critical positions during daily activities, they may not encompass the full spectrum of movements and forces experienced by individuals in real-world situations. The study's findings are therefore specific to the chosen boundary conditions and loading configurations.
⁃ Single-Patient Model and Weight Consideration: The geometric modeling was based on a knee replacement prosthesis suitable for a patient weighing approximately 80 kg. This single-patient model may not capture the variability in anatomical features and biomechanics across different individuals.

## 5 Conclusion

An optimized tibial model was proposed, featuring geometric alterations to the tray. Quantitative assessments demonstrated significant reductions in stress and strain, with von Mises stress decreasing by 23.35 % and equivalent strain by 17 % at a knee angle of 140°. Further evaluations at various angles, including 60°, consistently showed positive effects on stress and strain. These findings provide valuable insights into the mechanical behavior of knee prostheses and offer substantial evidence supporting the efficacy of linear material behavior assumptions. Our study introduces several unique aspects and innovations that distinguish it from existing research.

⁃ **Real-Life Prosthesis Modeling:** Unlike many studies that use simplified or generic prosthesis models, we utilized a meticulously constructed real-life knee replacement prosthesis model, developed using coordinate measuring and 3D scanning techniques. This high level of accuracy enhances the realism and applicability of our findings.
⁃ **Comprehensive Boundary Conditions and Material Assumptions:** We implemented and compared two distinct boundary conditions and both linear and nonlinear material assumptions in our finite element analysis. The minimal differences observed in stress error rates under these conditions provide insights into the efficiency of linear material behavior, optimizing computational resources.
⁃ **Focus on Rehabilitation Interventions:** Our study addresses a significant gap in the literature by investigating the dynamic stresses experienced by knee prostheses during rehabilitation interventions. This focus provides novel insights crucial for optimizing rehabilitation protocols and improving patient outcomes.
⁃ **Optimization of Tibial Model:** The proposed optimized tibial model incorporates geometric alterations to the tray, resulting in substantial reductions in von Mises stress (by 23.35 %) and equivalent strain (by 17 %) at a knee angle of 140°. This demonstrates the potential for design improvements to better accommodate mechanical demands during rehabilitation.
⁃ **Quantitative Assessments Across Different Angles:** Our detailed quantitative assessments at various knee angles provide a comprehensive understanding of knee prosthesis behavior under different loading conditions. This approach ensures that our findings are robust and applicable to a wide range of real-life scenarios.

In conclusion, this study underscores the importance of a detailed understanding of knee prosthesis behavior during rehabilitation. The quantitative results presented here, coupled with our novel modeling and analysis techniques, provide a solid foundation for refining existing prosthesis designs and guiding the development of next-generation knee prostheses. By incorporating these findings, it is possible to enhance the performance and durability of knee prostheses, ultimately benefiting patients undergoing total knee arthroplasty.

## CRediT authorship contribution statement

**Seyedhamidreza Emadiyanrazavi:** Writing – review & editing, Writing – original draft, Visualization, Validation, Supervision, Software, Resources, Project administration, Methodology, Investigation, Funding acquisition, Formal analysis, Data curation, Conceptualization. **Shahrokh Shojaei:** Writing – review & editing, Writing – original draft, Visualization, Validation, Supervision, Software, Resources, Project administration, Methodology, Investigation, Funding acquisition, Formal analysis, Data curation, Conceptualization.

## Declaration of competing interest

The authors declare that they have no known competing financial interests or personal relationships that could have appeared to influence the work reported in this paper.
